# Stress response of *Escherichia coli* to essential oil components – insights on low-molecular-weight proteins from MALDI-TOF

**DOI:** 10.1038/s41598-018-31255-2

**Published:** 2018-08-29

**Authors:** Matěj Božik, Pavel Cejnar, Martina Šašková, Pavel Nový, Petr Maršík, Pavel Klouček

**Affiliations:** 1 Czech University of Life Sciences, Faculty of Agrobiology, Food and Natural Resources, Department of Quality of Agricultural Products, Prague, Czech Republic; 2 University of Chemistry and Technology, Department of Computing and Control Engineering, Prague, Czech Republic; 3 Czech University of Life Sciences, Faculty of Agrobiology, Food and Natural Resources, Department of Plant Protection, Prague, Czech Republic

## Abstract

The antibacterial effects of essential oils and their components (EOCs) are usually attributed to effects on membranes and metabolism. Studies of the effects of EOCs on protein expression have primarily analysed proteins larger than 10 kDa using gel electrophoresis. In the present study, we used MALDI-TOF-MS to investigate the effects of EOCs on low-molecular-weight proteins. From 297 m/z features, we identified 94 proteins with important differences in expression among untreated samples, samples treated with EOCs, and samples treated with antibiotics, peroxide, or chlorine. The targets of these treatments obviously differ, even among EOCs. In addition to ribosomal proteins, stress-, membrane- and biofilm-related proteins were affected. These findings may provide a basis for identifying new targets of essential oils and synergies with other antibiotics.

## Introduction

Essential oils are mixtures of natural substances obtained from plants and have been used for years as antimicrobial agents^1^, in perfumes, and as natural food preservatives^2–4^. The antimicrobial effects of essential oils have been empirically proven in a number of studies^5–9^, but the mode of action of these substances has yet to be fully elucidated. Recent studies have revealed that the mechanisms of essential oils^10^ include degradation of the cell wall^11^, damage to bacterial membranes^12^, and reduction of the proton motive force and intracellular ATP^13^. In addition, sub-lethal concentrations of essential oils and their components (EOCs) alter the fatty acid composition of bacterial cell membranes^14,15^.

Essential oils can be useful in combatting bacterial strains that are resistant to other antimicrobials^16–19^. Synergistic effects of essential oils or EOCs with drugs or other natural substances have been reported^10,20,21^. These effects align with a recent front in the battle against bacterial resistance, that is, combining antibiotics with different targets in bacterial cells. This strategy is also referred to as “herbal shotgun” or “synergistic multi-target effects”^22^. Realising these mechanisms and avoiding multidrug resistant bacterial strains require the discovery of bioactive phytochemicals that target novel essential bacterial pathways. The development of omics sciences has allowed these mechanisms to be clarified at the molecular level, and previous studies have reported effects of essential oils and EOCs on the synthesis of some proteins^23–27^.

Most of the studies described above have used SDS PAGE^28–31^, 2-D gel electrophoresis (2D-GE)^32–36^ or liquid chromatography^37–40^ for protein separation and detection and MALDI-TOF-MS as the MS tool for protein identification. Proteomics analyses typically require time-consuming sample preparation steps^41^. Moreover, conventional 2D-GE is limited to the detection of denatured proteins in the size range of 10–200 kDa and is ineffective in distinguishing low-abundance proteins and extremely large or low-molecular-weight proteins (<10 kDa)^42,43^. MALDI-TOF-MS is a modern and fast alternative to traditional proteomic techniques that does not require lengthy sample preparation and can reliably identify changes in part of the bacterial proteome^37–39,44,45^. MALDI-TOF-MS is an important analytical method in the identification of proteins and evaluation of their role in biological processes. In *Escherichia coli*, MALDI-TOF-MS has been successfully used for comprehensive proteomics analyses of the stress response^45–47^. Bacterial MALDI-TOF-MS analysis can describe protein profiles up to 30 kDa, and characteristic mass spectral peaks are usually observed in the mass range from 3 kDa to 15 kDa. To observe higher masses, samples must be treated with surfactants^48^ and an appropriate matrix. The conventionally used matrices are α-cyano-4-hydroxycinnamic acid (HCCA), 2,5-dihydroxybenzoic acid (DHB) and sinapinic acid (SA)^41^. In this study, we focused on the effects of EOCs on the synthesis of low-molecular-weight proteins in *Escherichia coli* K12. The elucidation of the targets of these compounds in bacterial cells will provide a better understanding of their mechanisms of action and support the development of new strategies to combat bacterial pathogens.

## Results

### Minimum inhibitory concentrations

The antibacterial effects of the EOCs (see Table S1 for a complete list of the tested substances) were tested in three replicates, and the median MIC values were recorded (Table 1). Twelve of 61 compounds showed antibacterial properties within the tested concentrations and were further used as stress inducers in exponential-phase *E*. *coli*. Among these substances, the most active were eugenol (512 mg/L), carvacrol (256 mg/L), and trans-cinnamaldehyde (256 mg/L).

**Table 1:** Minimum inhibitory concentrations of tested compounds against *E. coli* by the microdilution method.

Compound^a^	CAS No.	MIC [mg·L^−1^]
Carvacrol	499-75-2	256
trans-Cinnamaldehyde	14371-10-9	256
Eugenol	97-53-0	512
(−)-Carvone	6485-40-1	1024
(+)-α-pinene	7785-70-8	1024
(±)-ß-Citronellol	106-22-9	1024
(1S)-(+)-3-Carene	498-15-7	1024
Thymol	89-83-8	1024
4-Carvomenthenol	562-74-4	2048
Citral	5392-40-5	2048
Geraniol	106-24-1	2048
Guaiacol	90-05-1	2048

^a^Substances included in stress-response analysis.

### Statistical analysis

**Figure 1:**
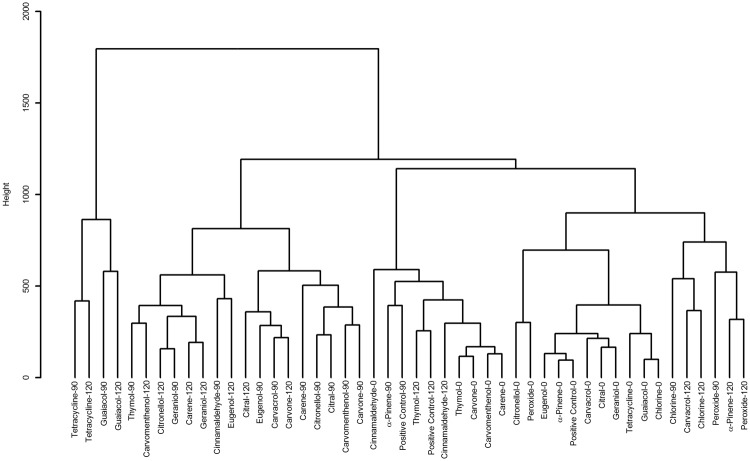
Dendrogram of principal component analysis scores for samples at time 0, 90 and 120 minutes. The data show six clusters with a critical distance level of 870: a cluster of tetracycline-treated samples, three clusters of terpene-treated samples, a cluster of untreated samples (time 0) and a cluster of oxidative agent-treated samples.

**Figure 2:**
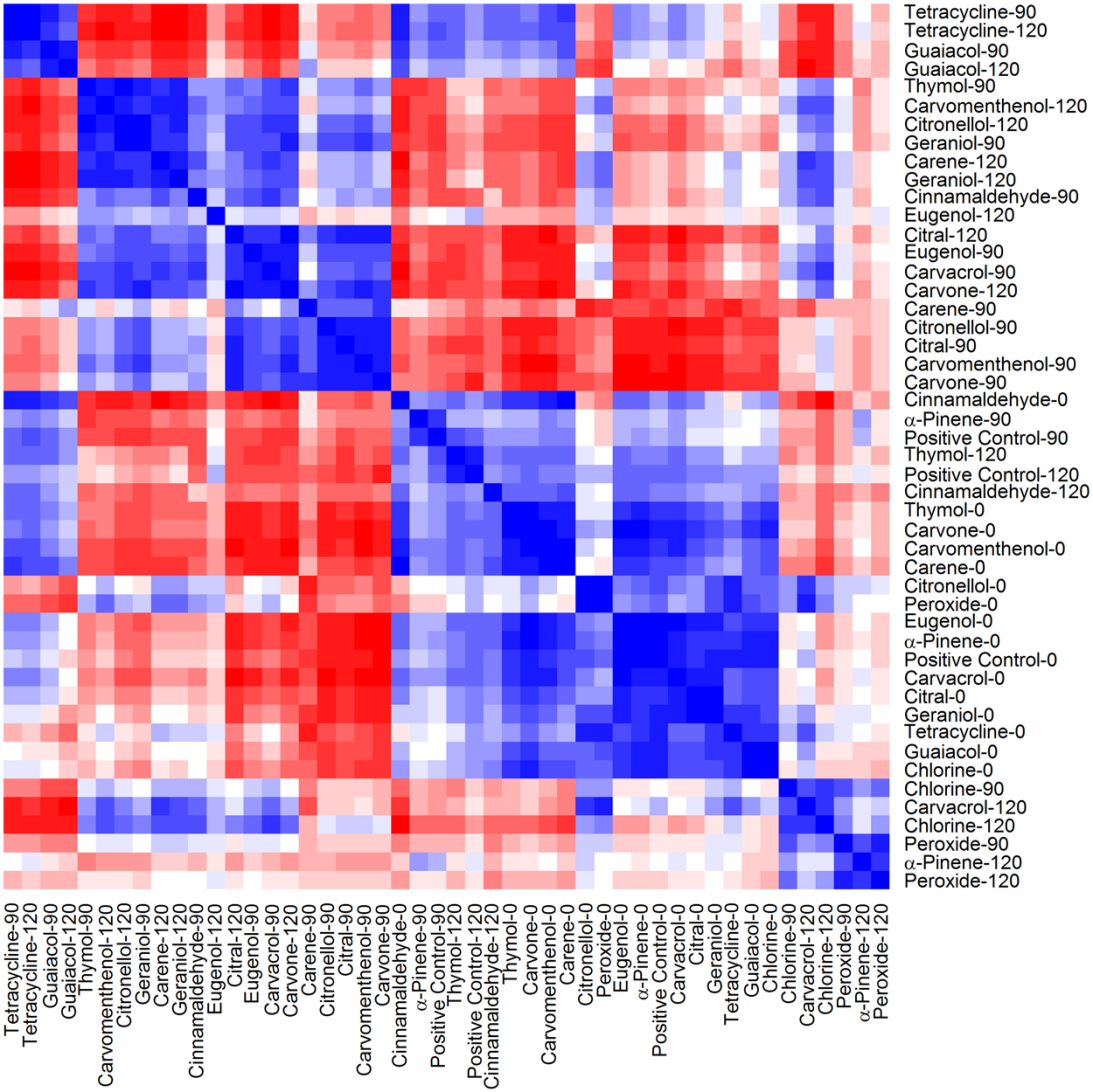
Correlation matrix of PCA scores of samples from time 0, 90 and 120 minutes. Positive correlations are shown in blue, and negative correlations are shown in red. The dark blue diagonal line indicates the intersection of identical samples. The diagram illustrates the similarity of the protein spectra of the samples before and after treatment.

The average spectra for each time and treatment (data available online: http://dx.doi.org/10.17632/s294p9sf9r.1) were analysed by principal component analysis (PCA, see S2 Figure), and dendrograms were created based on the PCA scores (Fig. 1). Samples from 0, 90 and 120 minutes were included. The dendrogram clearly revealed clusters corresponding to peroxide or chlorine treatment, tetracycline treatment, or untreated samples (0 minutes) and approximately three clusters of terpene-treated samples. The similarity of the spectra was then visualised as a correlation matrix (Fig. 2).

**Figure 3:**
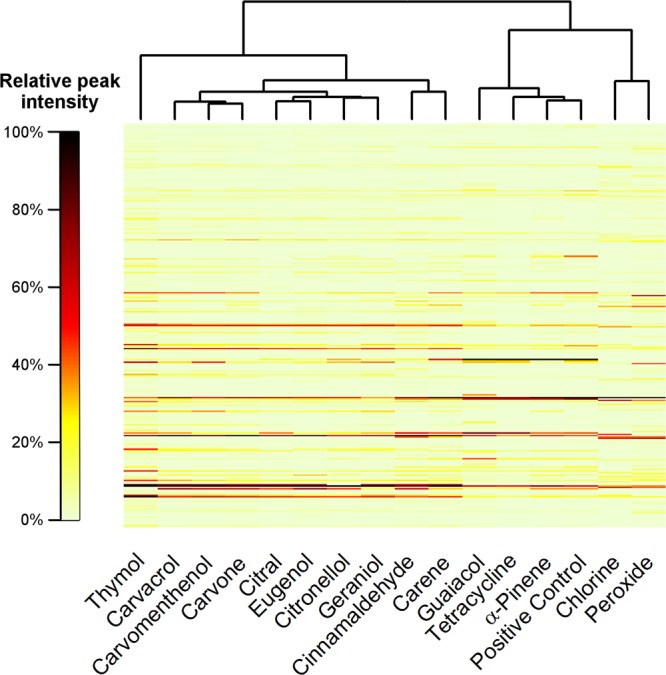
Treatment-clustered heat map of detected masses and their intensities. The heat map was constructed based on the normalised intensities of the detected m/z values.

mMass 5.5.0 was used for peak detection and peak list comparison^49^. Nearly 300 peaks with an intensity greater than 1% were detected in all samples after 90 minutes of treatment (Table S3). Approximately 97 peaks were detected per sample, and some were identified (Table S3) according to UniProtKB (www.uniprot.org). The intensities of different masses were visualised in a treatment-clustered heat map (Fig. 3).

### Protein identification

Unique m/z values were compared with the online databases UniProtKB/Swiss-Prot (release 2017_08) using the TagIdent tool (http://web.expasy.org/tagident/) and the literature^37–39,50^. Of the 297 detected m/z features, 94 were reliably identified according to UniProtKB/Swiss-Prot. The identified features are listed in Table S6 and include 25 ribosomal, 26 membrane-related, 9 cytosol- and DNA-related, and 4 stress indicators, with some overlap of functions. The functions and a complete list of m/z values are provided as supplementary material (Tables S3 and S4). Most of the remaining features are listed in the UniProtKB/TrEMBL database as uncharacterised proteins.

## Discussion

The antibacterial activities of carvacrol, trans-cinnamaldehyde, eugenol, carvone and α-pinene detected in this study (MICs of 256–1024 mg/L) were largely consistent with previous reports for various strains of *E*. *coli*^5,8,51–53^. However, for ß-citronellol, an MIC of 1024 mg/L was observed; values as low as 5 mg/L^11^ and as high as 4000 mg/L^54^ have been reported previously. For *Echinophora spinosa* essential oil, which contains 60% delta-3-carene, Glamočlija^55^ reported an MIC of 62 mg/L, which is significantly lower than our MIC for delta-3-carene. For some compounds, we obtained higher MICs than previously reported^8,51,52,56^. Most of these compounds are considered strong antimicrobials, but their effects depend greatly on their solubility in water^57,58^. Our results may have been influenced by the addition of Tween 80, although the concentration was rather low (0.5 % v/v). Guaiacol, despite its high MIC, is of interest because of its traditional use as well as its effect on *E*. *coli* proteins (see below). Guaiacol is a component of *Juniperus oxycedrus* L. essential oil and wood-tar creosote^59^ and is commonly used in antioxidant activity assays^60,61^. Historically, guaiacol was used as an antiseptic, and its derivative, guaifenesin, is used in over-the-counter drugs for respiratory tract infections. Guaiacol is listed under the name Seiro-gan in the Japanese Pharmacopoeia and is widely used for the treatment of diarrhoea^62^.

The cluster and correlation analyses revealed that the impacts of most of the EOCs on small *E*. *coli* proteins differ from those of antibiotics or chlorine and peroxide. The protein profile of the guaiacol treatment was closest to that of the antibiotic tetracycline. For both α-pinene and carvacrol, treatment for 120 minutes produced results similar to those for chlorine and peroxide treatment, whereas the results at 90 minutes of treatment with the same compounds were completely different and similar to those of the other EOCs. At longer treatment times, the results were generally not homogeneous, and the protein profiles for some of the EOCs, such as thymol and cinnamaldehyde, were similar to those of the untreated controls. The lack of an effect could be due to the use of exponential-phase bacterial cultures; the rapidly growing bacteria likely either overcame the effects of treatment (thymol, cinnamaldehyde) or were severely damaged to an extent similar to that observed for chlorine or peroxide treatment (α-pinene, carvacrol). Consequently, the 90-minute treatments were selected for detailed analysis of protein expression.

Conventional antibiotics target essential cellular processes, retarding growth and causing cell death. If bacteria are exposed to drugs at doses below that required to kill all bacteria in a population, antibiotic resistance can arise via natural selection of resistance-conferring mutations or gene transfer. In addition, at low doses, an effect known as hormesis may occur^63^. One strategy for combatting bacterial resistance is to employ a combination of different targets in bacterial cells, as represented by the new antibiotic teixobactin^64^. The effectiveness of a pair of synergistic antibiotics is greater than the sum of the efficacies of each antibiotic used alone^65^. Proteins with molecular weights of <25 kDa are involved in major biological processes such as ribosome formation, stress adaptation and cell cycle control^66^. Consequently, an easy and rapid screening method that specifically targets these proteins could be useful. Most previous studies have used SDS PAGE^28–31^, 2-D gel electrophoresis^32–36^ or LC^37–40^ for protein separation and detection and MALDI as the MS tool for protein identification. However, other studies have used MALDI as a comprehensive method for proteomics analyses of the *E*. *coli* stress response^45–47^. These experiments proved that MALDI-TOF could be used with online protein databases as a standalone tool for protein identification and semi-quantitation.

In our study, several differences in protein expression were detected between treated and untreated control samples. Some of these changes were shared among different groups of treatments: the uncharacterised protein YthA (m/z 4777,60, A8DYQ1) is typical of the stress response and was expressed more highly (intensity greater than 50%) in the treated samples than in the control (intensity 30%). The expression of this protein was lower in the samples treated with α-pinene, guaiacol and tetracycline, lowest in the chlorine-treated sample, and absent in the peroxide-treated sample. The high induction of the DNA-binding protein HU-alpha (m/z 9537,34, P0ACF0, hupA) in the samples treated with EOCs, namely citral, eugenol, geraniol, thymol, carvacrol, cinnamaldehyde, carvomenthenol, carvone and carene, is consistent with extreme environmental conditions. This protein stabilises DNA by wrapping to prevent its denaturation. *E*. *coli hupA hypB* double mutants that lack HU protein have severe cellular defects in cell division, DNA folding, and DNA partitioning^67^. The 30S ribosomal proteins S15 and S19 (m/z 10139,02, P0ADZ4, rpsO; m/z 10301,04, P0A7U3 rpsS) were moderately induced in EOC-treated samples. Both proteins are part of the cytosolic small ribosomal subunit and enable RNA binding. The rpsO operon is also induced during cold acclimation of cells and cold shock^68^. The induction of these proteins was lowest in the control and tetracycline-, guaiacol- and α-pinene-treated samples.

In addition to these similar changes in protein expression for most of the EOCs, several unique alterations of the protein spectrum were observed. Guaiacol induced the most distinct protein profile: UPF0434 protein (m/z 6960.26, P64614, YcaR) and 23S rRNA methylase leader peptide (Erythromycin resistance leader peptide, m/z 3802.46, P10739, ermC) were detected only in the guaiacol-treated sample at very high levels. The former protein is involved in biofilm formation, an important antibacterial target, whereas the latter is involved in the regulation of the synthesis of the erythromycin resistance protein^69^. Guaiacol has been shown to inhibit biofilm formation by *E*. *coli*^70^. Eugenol specifically induced the prophage outer membrane lipoprotein RzoR (m/z 4586.76, P58042, rzoR), which is involved in viral disruption of the outer membrane during virus exit. *YthA* (m/z 4777,60, A8DYQ1) and *YoaJ* (m/z 2690,98, C1P603) are among the short essential genes of *E*. *coli* that are involved in the cell’s response to stress, such as heat shock, cold shock, cell envelope stress, oxidative stress, thiol stress and acid stress^42,71^. Another uncharacterised lipoprotein, YqhH (m/z 7306,22, P65298), was detected only in the peroxide- and chlorine-treated samples. YqhH is part of the outer cell membrane and functions as a lipid-anchor peptidoglycan-anchor, consistent with the strong antibacterial effects of chlorine and peroxide, which disturb bacterial membranes. Another change in protein expression specific to the chlorine- and peroxide-treated samples was that of 50S ribosomal protein L35 (m/z 7290,90, B1XG24), which is a structural constituent of the ribosome. This protein was also observed in cinnamaldehyde- and carene-treated samples. The lower amounts of stationary phase-induced ribosome-associated protein (m/z 5096,84, P68191, SRA) in the tested samples indicate retarded cell development in the treated samples compared with the control. SRA is an integral part of the ribosome, especially of the 30S subunit in stationary and, to a lesser extent, exponential phase^72^. 50S ribosomal protein L30 (m/z 6411,04, P0AG51, rpmD) was highly induced by thymol, carvacrol and cinnamaldehyde. This protein is structural constituent of the ribosome and functions in the aggregation, arrangement and bonding of constituent RNAs and proteins to form the large ribosomal subunit. In *E*. *coli*, the *rpmD* and *lolD*, *rplC*, and *rpoB* genes are essential for cell growth^73^. Thymol also intermediately increased the expression of the putative uncharacterised protein b0309 (m/z 7851,78, P75688), which was also detected in the other EOC-treated samples but completely missing in the control. The gene encoding this protein was induced in experiments with *E. coli* K12 upon deletion of the *ydgG* gene, which encodes a protein that is induced in *E*. *coli* biofilms and influences resistance to several antimicrobials^74^.

In conclusion, we identified 94 proteins among 297 m/z features and important differences in expression patterns among untreated, EOC-treated, antibiotic-treated and peroxide- and chlorine-treated samples. Although the analysis of low-molecular-weight proteins does not provide a complete picture of the proteome, it represents a piece of the puzzle that is commonly missed by most proteomics methods. Our results not only support previous theories on the impact of EOCs on bacterial membranes and the cell wall but also provide evidence of different mechanisms of action leading to diverse bacterial responses. The most interesting EOCs likely influence biofilm formation, resistance to antibiotics, and ribosomal functionality; these types of effects have previously been observed for some EOCs^13,25,54,70,75^. Moreover, the feasibility of direct MALDI-TOF analysis of the bacterial stress response was demonstrated. The observed effects of EOCs on low-molecular-weight proteins could reveal new targets for screening by this relatively rapid method and contribute to the knowledge of the antibacterial mode of action of EOCs. Given the acknowledged synergy of essential oils with other antibiotics, our approach could be useful in combatting antibacterial resistance. Further studies are needed to test this hypothesis.

## Material and Methods

### Essential oil components

All essential oil components (Table S1) were obtained from a commercial supplier Sigma-Aldrich (DE) and stored in air-tight sealed glass vials or the original bottles at 4 °C.

### Minimal inhibitory concentration

*Escherichia coli* (DSM 18039) was obtained from the German Collection of Microorganisms and Cell Cultures GmbH. Bacterial stock cultures were maintained in Mueller-Hinton Broth (Oxoid, CZ) with 50% glycerine at −80 °C. Working cultures of the bacterial strain were grown in LB Broth - Lennox (Sigma-Aldrich, DE) at 37 °C for 24 hours before the tests. An inoculum was then created by dilution in PBS (phosphate-buffered saline) to a final cell concentration of 10^8^ CFU/mL, which was confirmed by density measurement in McFarland units (densitometer McFarland type DEN-1B, Biosan, LV). A modification of the EUCAST microdilution method (EUCAST, 2003) was used for antimicrobial testing. A two-fold serial dilution of EOCs ranging from 2048 to 64 mg/L was prepared for all tested substances in LB broth with 1% Tween 80 in 96-well microtitration plates. The plates were inoculated by pin replicator and incubated at 37 °C overnight. After incubation, the minimum inhibitory concentrations (MICs) were recorded. MICs were expressed as the lowest concentration at which a compound inhibited visible bacterial growth. The microdilution assay was performed in triplicate, and the resulting median MICs were recorded.

### Bacterial stress response in exponential phase

Microtitre plate wells were filled with 100 µL of LB broth – Lennox and then inoculated with a fresh inoculum prepared from an overnight culture of *E*. *coli*. The plates were incubated at 37 °C for 5 hours, which corresponds to the end of lag phase. The stock solutions of the active substances from the MIC assay (Table 1) were diluted to 2x MIC in LB broth – Lennox, and 100 μL of this the dilution was added to the *E*. *coli* culture. As reference stress conditions, tetracycline 5 mg/L^76^, NaClO 5 mg/L and hydrogen peroxide 3 g/L^77^ were used. Untreated cultures cultivated under the same conditions were used as controls. At 0, 30, 60, 90 and 120 minutes after treatment, the cultures were transferred from wells to Eppendorf tubes and mixed with 1 mL of PBS, and the cells were harvested by centrifugation at 20,000 × g for 2 minutes in a Rotanta 450 R (Hettich, DE). The supernatant was discarded, and the pellets were resuspended in 1 mL of 70% ethanol in an Eppendorf tube. The cells were then prepared for MALDI-TOF analysis according to the standard Bruker procedure. The samples were centrifuged at 20,000 × g for 2 minutes, and the supernatant was decanted. After centrifugation for an additional two minutes, the residual ethanol was removed from the pellets by pipette. The samples were left to dry for 30 minutes at room temperature to increase the extraction efficiency. Then, 5 μL of 70% formic acid (Sigma-Aldrich, DE) was added to the pellet and mixed thoroughly by vortexing. The same volume of ultra-pure acetonitrile (Fluka, DE) was added to the tube and mixed again. Finally, the samples were centrifuged at 20,000 × g for 2 minutes, and 1 μL of supernatant was transferred onto an MTP 384 ground steel BC target plate (Bruker, DE). Each sample was placed on one spot around a spot with calibrant (BTS, Bruker, DE). Once the sample spots dried, the samples were immediately overlaid with 1 μL of HCCA (α-cyano-4-hydroxycinnamic acid, Bruker, DE) matrix solution (acetonitrile 50%, water 47.5% and trifluoracetic acid 2.5%; 10 mg/mL HCCA). The sample spots were allowed to dry before analysis. MALDI-TOF analysis was performed using an Autoflex Speed (Bruker, DE) and flexControl 3.4 (Build 135) with a modified MALDI Biotyper method (MBT_FC). Ion source 1 was 19.38 kV, ion source 2 was 18.18 kV, and detection was set from 1000 to 15,500 Da. All experiments were performed in independent biological triplicates for each agent and treatment time. For each sample, three spectra were measured, and each spectrum was collected by 4000 shots in 200 steps.

### Statistical analysis of the stress response

Due to its limitations, MALDI Biotyper Version 3.1 could not be used to analyse the stress response. Instead, the public package R-project multiMS-toolbox and Mass Spectrum Analysis and Data Conversion Tool^78,79^ were used.

The mass spectra of each sample treated with the tested agent from all three replicates and the measured dots were exported using FlexAnalysis 3.4 Compass 1.4 (Bruker Daltonics, Bremen, Germany). All spectra were preprocessed using a customised version of multiMS-toolbox 2.08 run in R software 3.4.2. All exported mass spectra were pre-processed by a Savitzky-Golay smoothing filter, and the best-matched exponential baseline was removed. The signal intensities were normalised to the same median intensity ratios. The subsequent PCA of the full-spectrum data was evaluated using multiMS-toolbox 2.08 and R software 3.4.2. The results were subsequently confirmed by PCA of the averaged spectra of all samples of the same treatment and the same time (averaged over biological replicates and all three measured spectra) and by PCA of the extracted and matched peak intensity data.

Hierarchical cluster analysis was performed by using the ‘hclust’ package of R software 3.4.2 on peak intensities extracted from averaged spectra of the treated samples at 90 minutes and 120 minutes. The distance matrix was computed using the Manhattan method on the PCA-transformed score space (all components explaining at least 98.8% of the cumulative variance were included) of the averaged full-spectrum data. Hierarchical clustering was performed with the completed linkage method. The correlation matrix was computed using Pearson’s correlation coefficients of the PCA scores of the averaged full-spectrum data. The samples in the matrix were then reordered according to the identified hierarchical clustering.

The heat map of the detected masses and intensities (Table S5) was constructed from peak intensities extracted from the averaged pre-processed spectra of the 90-minute treated samples, and their distance matrix was computed using the Manhattan method. Hierarchical clustering was computed using the completed linkage method. The samples in the heat map were reordered according to the identified hierarchical clustering. The extracted mass lists with m/z (mass-to-charge ratio) values were compared with the online databases UniProtKB/Swiss-Prot (release 2017_08) using the TagIdent tool^80^, which is freely accessible online (https://web.expasy.org/tagident/), and with previously reported results^37–39,50^. The search criteria were as follows: protein MW Da ± 0.03%, taxonomy *Escherichia coli*.

### Quality control

The Bruker bacterial test standard is an *E*. *coli* extract spiked with two high-molecular-weight proteins. The standard was developed by Bruker for quality control of the MALDI Biotyper system. The specific composition of the standard covers the entire mass range of proteins used in our MALDI method. There are 8 described proteins, and their m/z values have an accuracy of 300 ppm. These proteins were used as standards for calibration. Eight sample spots were placed around the calibrant to obtain the best available accuracy. The measured spectra were visually inspected for spectrum quality control using FlexAnalysis software, and defects were excluded from analysis.

## Electronic supplementary material

Supplementary Information

S3 Table

S4 Table

## Data Availability

The datasets generated and/or analysed during the current study are available in the Mendeley Data repository at 10.17632/s294p9sf9r.1.
