# Supplementary material for: Stress response of Escherichia coli to essential oil components – insights on low-molecular-weight proteins from MALDI-TOF
Source: Sci Rep. 2018 Aug 29;8:13042. doi: 10.1038/s41598-018-31255-2 (PMC6115441; doi:10.1038/s41598-018-31255-2)

Stress response of *Escherichia coli* to essential oil components – insights on low-molecular-weight proteins from MALDI-TOF

Matěj Božik[1], Pavel Cejnar[2,3], Martina Šašková[1], Pavel Nový[1], Petr Maršík[1], Pavel Klouček[1*]

[1] *Czech University of Life Sciences, Faculty of Agrobiology, Food and Natural Resources, Department of Quality of Agricultural Products, Prague, Czech Republic*

[2] *University of Chemistry and Technology, Department of Computing and Control Engineering, Prague, Czech Republic*

[3] *Czech University of Life Sciences, Faculty of Agrobiology, Food and Natural Resources, Department of Plant Protection, Prague, Czech Republic*

∗ Corresponding author.

E-mail address: kloucek@af.czu.cz

Supplementary Table S1

Minimum inhibitory concentrations of tested compounds against *E. coli* by the microdilution method

Compound	CAS No.	MIC [µL/L]
Carvacrol[a]	499-75-2	256
trans-Cinnamaldehyde[a]	14371-10-9	256
Eugenol[a]	97-53-0	512
(−)-Carvone[a]	6485-40-1	1024
(+)-α-pinene[a]	7785-70-8	1024
(±)-ß-Citronellol[a]	106-22-9	1024
(1S)-(+)-3-Carene[a]	498-15-7	1024
Thymol[a]	89-83-8	1024
4-Carvomenthenol[a]	562-74-4	2048
Citral[a]	5392-40-5	2048
Geraniol[a]	106-24-1	2048
Guaiacol[a]	90-05-1	2048
4-Allylanisole	140-67-0	>2048
Allyl disulfide	2179-57-9	>2048
trans-Anethole	4180-23-8	>2048
Anisole	100-66-3	>2048
(−)-Bornyl acetate	5655-61-8	>2048
(−)-α-Bisabolol	23089-26-1	>2048
Butyl isothiocyanate	592-82-5	>2048
ß-Caryophyllene	87-44-5	>2048
(−)-trans-Caryophyllene	87-44-5	>2048
Cedrol	77-53-2	>2048
Cineole	470-82-6	>2048
(±)-Citronellal	106-23-0	>2048
Citronellyl propionate	141-14-0	>2048
p-Cymene	99-87-6	>2048
2-Decanol	1120-06-05	>2048
D-Dihydrocarvone	7764-50-3	>2048
Farnesene	mixture of isomers	>2048
(+)-Fenchone	4695-62-9	>2048
Geranyl acetate	105-87-3	>2048
Hexyl hexanoate	6378-65-0	>2048
(−)-Isopulegol	89-79-2	>2048
(R)-(+)-Limonene	5989-27-5	>2048
(-)-Linalool	126-91-0	>2048
(-)-Menthone	14073-97-3	>2048
(-)-β-Pinene	18172-67-3	>2048
α-Phellandrene	99-83-2	>2048
(+)-Rose oxide	16409-43-1	>2048
α-Terpinene	99-86-5	>2048
γ-Terpinene	99-85-4	>2048
Terpinolene	586-62-9	>2048

[a] substances included in stress response analysis

Supplementary Figure S2

Principal component analysis of the average spectra for each time and treatment – samples scores plot for PC1×PC2 projection (top) and PC1×PC3 projection (bottom). Samples from 0, 90 and 120 minutes were included.

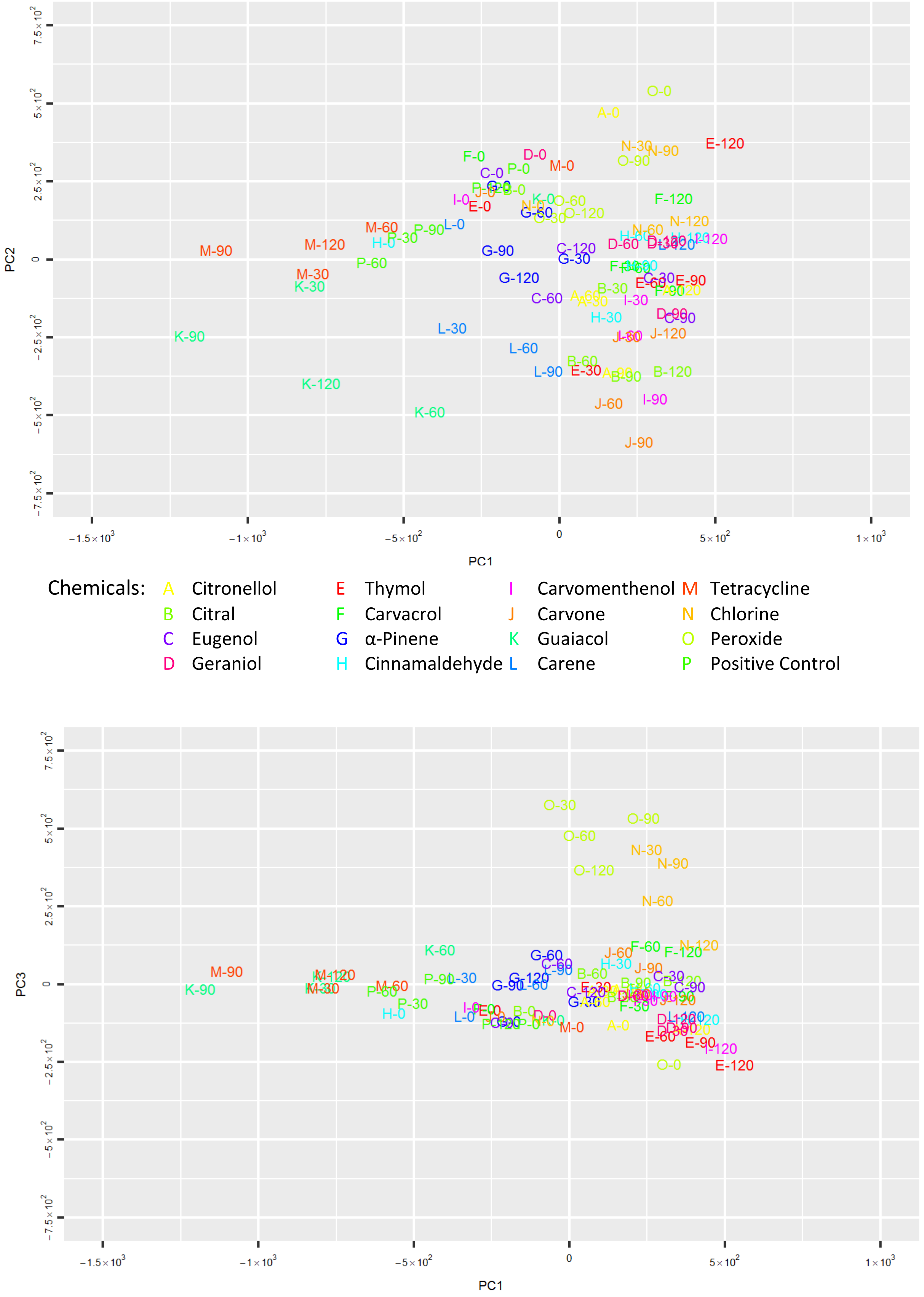

Supplement: Supplementary file 1 — Supplementary Information [file 41598_2018_31255_MOESM1_ESM.pdf]
